# An Unusual Cause of Intestinal Obstruction: Internal Supravesical Hernia

**DOI:** 10.5334/jbsr.2020

**Published:** 2020-02-03

**Authors:** Jin-A Ryoo, Seung Soo Kim

**Affiliations:** 1 Department of Radiology, Soonchunhyang University College of Medicine, Cheonan Hospital, Cheonan-si, KR

**Keywords:** Hernia, Intestinal obstruction, Multidetector computed tomography

## Abstract

**Teaching Point:** The typical CT finding of internal supravesical hernia is a herniated and dilated bowel loop beneath a compressed urinary bladder.

## Case History

**Figure 1:**
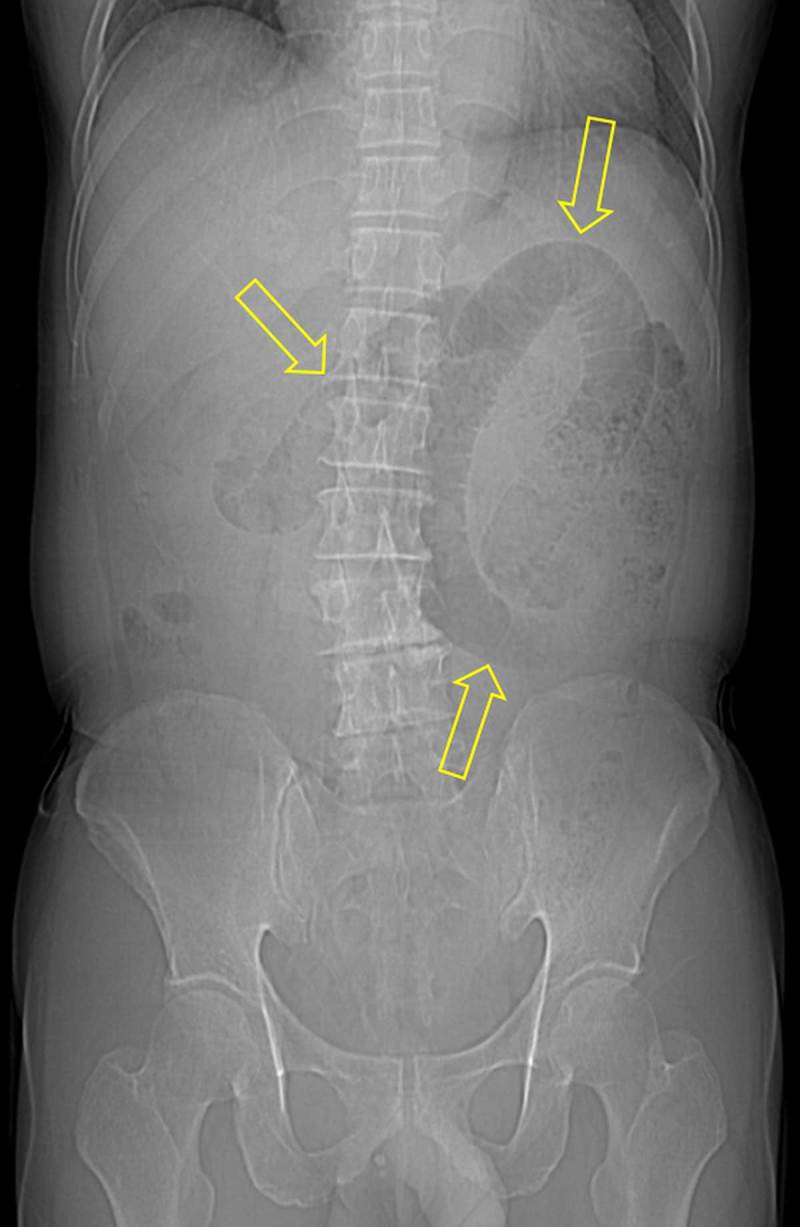


**Figure 2:**
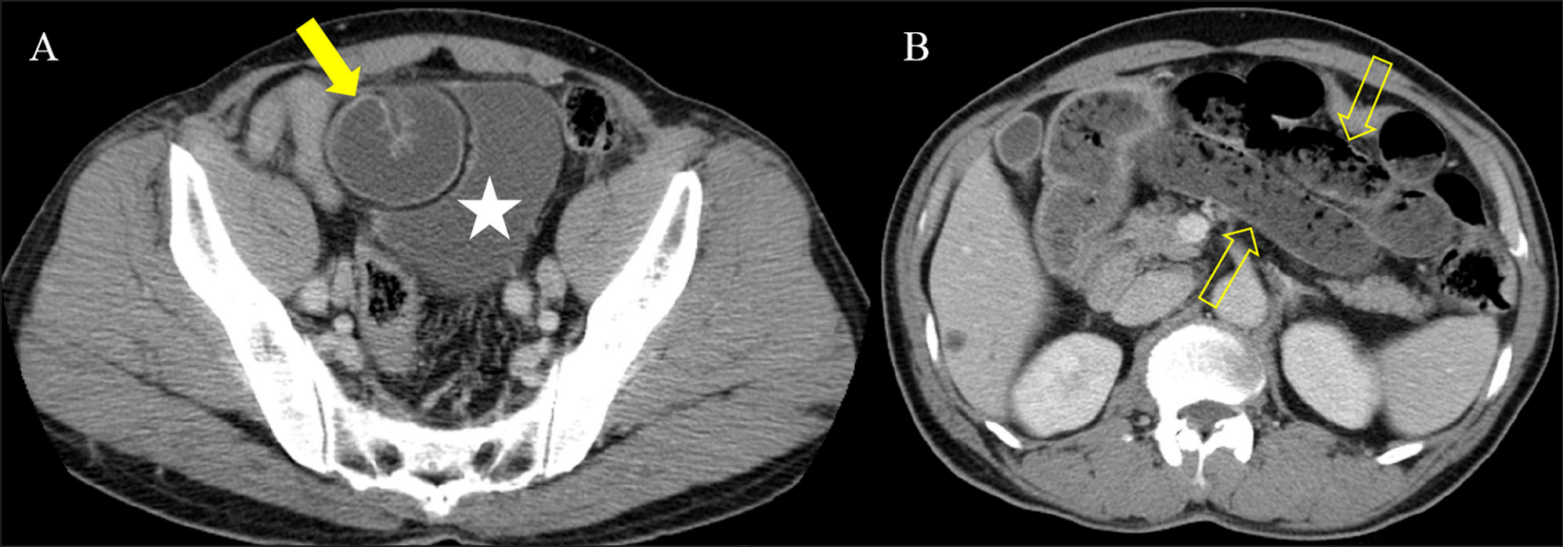


**Figure 3:**
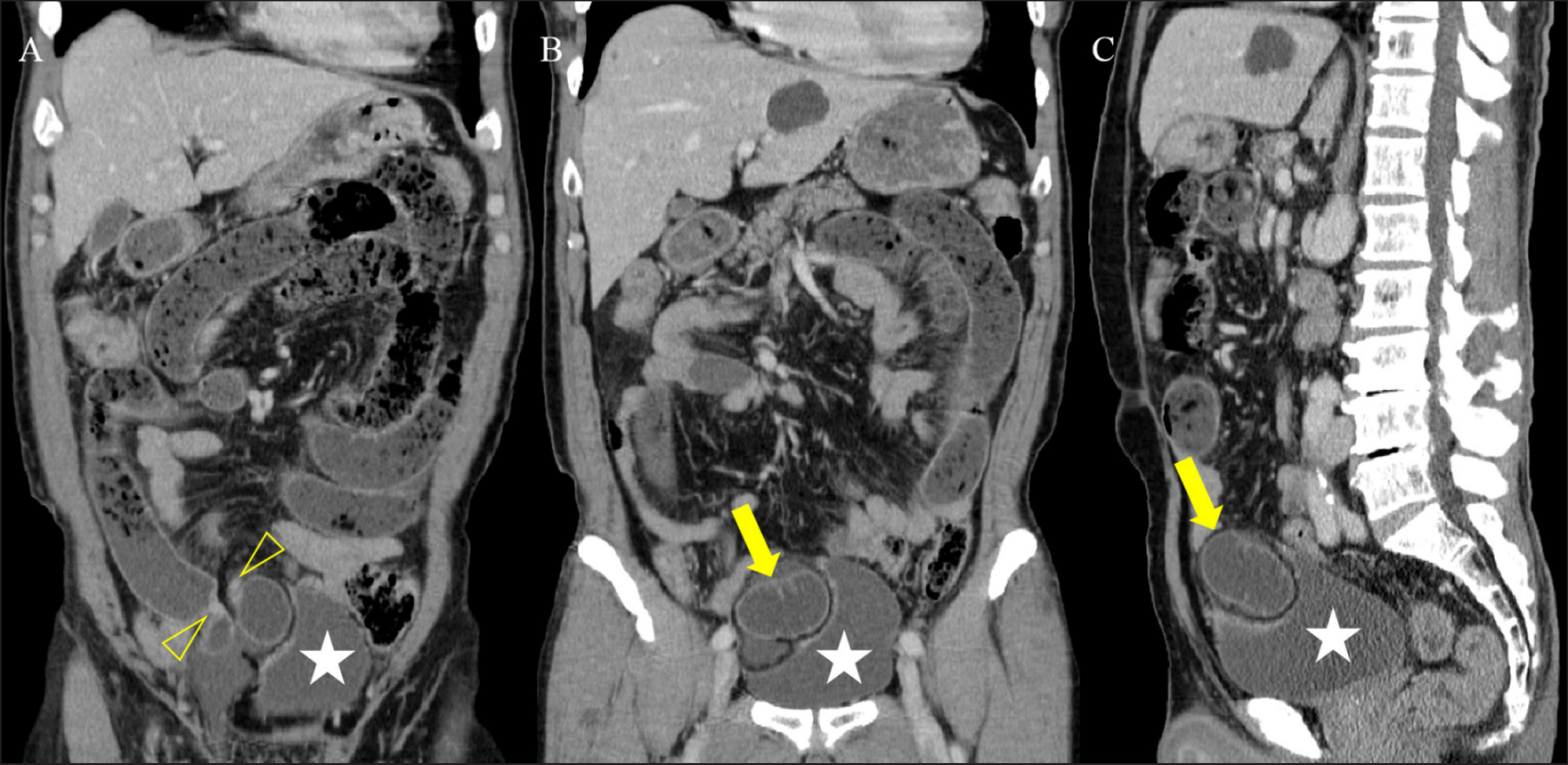


A 64-year-old man presenting with lower abdominal pain was admitted to our hospital. He had a history of herniorrhaphy for right inguinal hernia 20 years earlier. All laboratory results were within normal limits. Contrast-enhanced computed tomography (CT) was performed for evaluation of abdominal pain, and CT scanogram (Figure 1) showed dilated small bowel loops (open arrows). Axial CT images (Figure 2) demonstrated a U-shaped, fluid-filled bowel loop (arrow) that pressed the urinary bladder (white star), and the proximal small bowel loops (open arrows) were dilated. Coronal reformatted CT images (Figure 3A and 3B) identified two transition points (open arrowheads) of the pseudo-encapsulated small bowel loop (arrow) above the urinary bladder (white star). On sagittal reformatted CT image (Figure 3C), an incarcerated bowel loop (arrow) compressed the anterior wall of the urinary bladder (white star). The patient underwent surgery and was diagnosed with intestinal obstruction caused by internal supravesical hernia (SVH).

## Comment

SVH is a rare type of hernia that occurs at the supravesical fossa, a space bound laterally by the medial umbilical ligament, medially by the median umbilical ligament, and inferiorly by the peritoneal reflection between the anterior abdominal wall and the dome of the urinary bladder. SVH is divided into internal and external types. When herniated bowel protrudes through the supravesical fossa to the anterior abdominal wall, it is classified as an external type. On the other hand, intestine that herniates downward through the supravesical fossa into a space around the urinary bladder is classified as an internal type [1]. A previous report proposed the subtypes of internal SVH as anterior, posterior, and right or left lateral type depending on position of the herniated sac relative to the urinary bladder [1].

CT is a reliable imaging modality for diagnosis of SVH. The characteristic CT finding of internal SVH is a herniated bowel loop beneath a compressed urinary bladder. The herniated intestine usually shows a sac-like appearance, which is a common imaging feature of an internal hernia. Internal hernias, including internal SVH, frequently cause closed-loop obstruction and subsequent bowel strangulation. Therefore, surgical reduction is needed in patients with internal SVH [1].
